# Microflora of the penile skin-lined neovagina of transsexual women

**DOI:** 10.1186/1471-2180-9-102

**Published:** 2009-05-20

**Authors:** Steven Weyers, Hans Verstraelen, Jan Gerris, Stan Monstrey, Guido dos Santos Lopes Santiago, Bart Saerens, Ellen De Backer, Geert Claeys, Mario Vaneechoutte, Rita Verhelst

**Affiliations:** 1 Department of Obstetrics & Gynaecology, Ghent University Hospital, Ghent, Belgium; 2 Department of Plastic Surgery, Ghent University Hospital, Ghent, Belgium; 3 Department of Clinical Chemistry, Microbiology & Immunology, Ghent University Hospital, Ghent, Belgium; 4 Laboratory of Bacteriology Research, Department of Clinical Chemistry, Microbiology & Immunology, Ghent University Hospital, Ghent, Belgium

## Abstract

**Background:**

The microflora of the penile skin-lined neovagina in male-to-female transsexuals is a recently created microbial niche which thus far has been characterized only to a very limited extent. Yet the knowledge of this microflora can be considered as essential to the follow-up of transsexual women. The primary objective of this study was to map the neo-vaginal microflora in a group of 50 transsexual women for whom a neovagina was constructed by means of the inverted penile skin flap technique. Secondary objectives were to describe possible correlations of this microflora with multiple patients' characteristics, such as sexual orientation, the incidence of vaginal irritation and malodorous vaginal discharge.

**Results:**

Based on Gram stain the majority of smears revealed a mixed microflora that had some similarity with bacterial vaginosis (BV) microflora and that contained various amounts of cocci, polymorphous Gram-negative and Gram-positive rods, often with fusiform and comma-shaped rods, and sometimes even with spirochetes. *Candida *cells were not seen in any of the smears.

On average 8.6 species were cultured per woman. The species most often found were: *Staphylococcus epidermidis*, *Streptococcus anginosus *group spp., *Enterococcus faecalis, Corynebacterium *sp., *Mobiluncus curtisii *and *Bacteroides ureolyticus*. Lactobacilli were found in only one of 30 women

There was no correlation between dilatation habits, having coitus, rinsing habits and malodorous vaginal discharge on the one hand and the presence of a particular species on the other. There was however a highly significant correlation between the presence of *E. faecalis *on the one hand and sexual orientation and coitus on the other (p = 0.003 and p = 0.027 respectively).

Respectively 82%, 58% and 30% of the samples showed an amplicon after amplification with *M. curtisii*, *Atopobium vaginae *and *Gardnerella vaginalis *primer sets.

**Conclusion:**

Our study is the first to describe the microflora of the penile skin-lined neovagina of transsexual women. It reveals a mixed microflora of aerobe and anaerobe species usually found either on the skin, in the intestinal microflora or in a BV microflora.

## Background

Gender identity disorder (GID) is a condition in which a person identifies as belonging to the opposite gender as the one he or she was birthed to and whereby this person feels significant discomfort about this condition. Transsexualism is considered as the most extreme form of gender identity disorder [1] and will most typically require sex reassignment surgery (SRS) following the Standards of Care of the World Professional Association of Transgender Health (WPATH), formerly known as the 'Harry Benjamin Gender Dysphoria Association' (HBIGDA) [2]. In male-to-female transsexual patients, also called 'transsexual women', this SRS consists of removal of the male reproductive organs (testes and penis), creation of a neovagina (vaginoplasty) and -clitoris and, in most patients, implantation of breast prostheses. Since the start of the gender team at our institution (Ghent University Hospital) we performed SRS in more than 400 male-to-female transsexual individuals. For the creation of the neovagina in transsexual women we use the technique of the inverted penile skin flap to line a newly created space between the prostate-bladder and the rectum. This technique is nowadays the standard technique for creation of the vagina in transsexual patients [3].

Under normal conditions, the lower female genital tract harbors a commensal microflora that primarily consists of lactobacilli which confer antimicrobial protection to the vagina. In addition, under adequate vaginal estrogen levels, the vaginal epithelium and its associated mucous layers help to regulate and support the intrinsic bacterial and mucosal defense system [4]. However, in case the vaginal hydrogen peroxide producing lactobacilli fail to sustain, an overgrowth by other bacteria occurs, as is most typically observed with commensal bacterial vaginosis-associated micro-organisms [5]. These commensals include *Gardnerella vaginalis*, *Atopobium vaginae*, *Prevotella *spp., anaerobic Gram-positive cocci, *Mobiluncus *spp. and *Mycoplasma hominis*.

While the composition of the normal vaginal microflora (VMF) has been extensively studied by conventional culture techniques and molecular methods [6,7], thus far, there is no information in the literature on the vaginal microflora in transsexual women treated with the technique of the inverted penile skin flap. The single report on the microflora of the neo-vagina concerned 15 patients who were treated with pedicled intestinal (sigmoid) transplants [8].

Yet, knowledge of the VMF in transsexual women can be considered as essential to ensure proper follow-up of the women, e.g. in case they present with vulvar or vaginal complaints (pain, odour, itch, etc) or in case of overt genital inflammation and/or infection.

The primary objective of this study was to map the VMF in a group of transsexual patients treated with the inverted penile skin technique. Secondary objectives were to describe possible correlations of this microflora with multiple patients' characteristics, such as sexual orientation, the incidence of vaginal irritation and malodorous vaginal discharge.

## Results

### General characteristics

The mean age of the transsexual women who participated in the study was 43.1 years (SD = 10.4) and the mean time elapsed since sex reassignment surgery – herewith denoted by vaginoplasty – was 6.3 years (SD = 6.4).

The vast majority of participants were taking oestrogen replacement therapy (47/50), with three women not taking any oestrogens since they were at increased thrombo-embolic risk. In addition to daily oestrogen substitution two women also administered continuously antiandrogens (cyproterone acetate 10 mg daily).

### Hormonal status

Median serum levels for testosterone (ng/dl) and oestradiol (pg/ml) were 29.57 (interquartile (IQ) range 21.45–38.24) and 49.13 (IQ range 28.61–96.17) respectively.

### Sexual and genital characteristics

About half of the transsexual women (54%) were involved in a steady relationship at the time of the survey. Forty-four percent of the transsexual women indicated heterosexual orientation (n = 22), 22% reported homosexual preference (n = 11), 28% had a bisexual orientation (n = 14) and the remainder of women (n = 3) identified themselves as 'not sexually interested' (6%).

Eleven women (22%) had regular episodes of vaginal irritation while nine (18%) frequently experienced dysuria. There was a significant correlation between having episodes of vaginal irritation and dysuria (0.505, p < 0.001). Thirty-four out of the 50 patients answered the additional questions about the use of vaginal products and presence of bad smelling discharge. Nineteen out of these 34 women (55.9%) reported regular use of vaginal hygiene products. Ten of them were using a iodine solution (Isobetadine Gynecological solution, Meda Pharma, Brussels, Belgium), 7 used a solution with low pH containing lactic acid and milk serum (different manufacturers), one was using a body douche gel and another applied plain tap water.

Eight out of 34 (23.5%) had frequent episodes of bad-smelling vaginal discharge. There was no correlation between malodorous vaginal discharge and vaginal irritation. Likewise there was no correlation between vaginal rinsing habits and the vaginal pH and malodorous vaginal discharge.

### Vaginal examination and microflora

A normal sized speculum (2.5 cm wide) could be used in 74% of women (37/50), whereas the smaller type (2.0 cm wide) had to be used in the remainder of women. Only in one patient insertion of a speculum was impossible due to almost complete obliteration of the vagina. Although this was not a study criterion and therefore not scored, a foul smell of the vagina was observed in most patients.

The mean vaginal pH was 5.88 (SD = 0.49, range 5.0–7.0). There was no correlation between the vaginal pH and complaints of irritation, dysuria or malodorous discharge.

#### Gram stain

**Figure 1:**
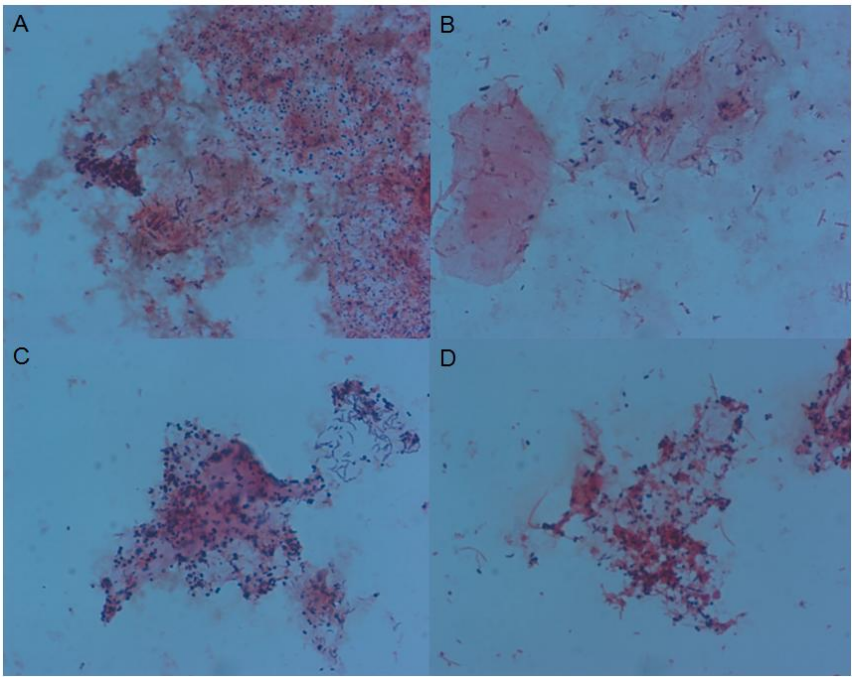
**Microscopic image (1000×) of Gram-stained neovaginal smears illustrating the observed diversity: various amounts of cocci (A), polymorphous Gram negative and Gram positive rods, often with fusiform (B) and comma-shaped rods (C), and sometimes even with spirochetes (D)**.

The fifty neovaginal swab specimens were Gram stained. For six smears, one with numerous white blood cells, few bacteria were found. Forty-four smears revealed mixed microflora that had some similarity with bacterial vaginosis microflora and that contained various amounts of cocci, polymorphous Gram-negative and Gram-positive rods, often with fusiform and comma-shaped rods, and sometimes even with spirochetes (Figure 1). In five of these smears white blood cells were seen. *Candida *cells were not seen in any of the smears. There was no correlation between malodorous vaginal discharge and painful dilation on one hand and the presence of leucocytes on Gram stain on the other hand.

#### Identification of cultured isolates from 30 transsexual women by tDNA-PCR and 16S rRNA gene sequencing

Of the 582 isolates that were picked after culture of the 30 neovaginal specimens on 5 different media, a total of 378 isolates could be identified by tDNA-PCR. A further 56 isolates could be identified after sequencing of the 16S *rRNA *gene. 79 different species and 12 possibly novel species (referred to as TSW Genotype A to L) were identified (Table 1). TSW Genotype B, I and K had more than 98% similarity to previously cultured isolates. All other genotypes had between 83% and 99% similarity with previously cloned sequences (Table 1).

**Table 1:** Detailed composition of the neovaginal microflora of 30 swab samples, as determined by culture and tDNA-PCR based identification.

Cultured species	n	Cultured species	n
**Actinobacteria**		**Firmicutes**	
*Actinobaculum massiliense*	2	*Anaerococcus hydrogenalis*	1
*Actinobaculum schaalii*	1	*Anaerococcus tetradius*	1
*Actinomyces meyeri*	6	*Anaerococcus vaginalis*	3
*Actinomyces neuii*	2	*Bacillus *sp.	1
*Actinomyces radingae*	1	*Clostridium perfingens*	1
*Actinomyces *sp.	2	*Enterococcus faecalis*	13
*Actinomyces turicensis*	1	*Enterococcus *sp.	1
*Actinomyces urogenitalis*	2	*Facklamia hominis*	1
*Arcanobacterium bernardiae*	1	*Finegoldia magna*	7
*Arcanobacterium pyogenes like*	1	*Lactobacillus casei*	1
*Atopobium vaginae*	2	*Peptoniphilus indolicus*	6
*Bifidobacterium bifidum*	1	*Peptoniphilus lacrimalis*	6
*Bifidobacterium longum*	1	*Peptoniphilus *sp.	6
*Corynebacterium aurimucosum*	2	*Staphylococcus aureus*	3
*Corynebacterium glucuronolyticum*	1	*Staphylococcus capitis*	1
*Corynebacterium pseudogenitalium*	1	*Staphylococcus cohnii*	1
*Corynebacterium *sp.	13	*Staphylococcus epidermidis*	19
*Gardnerella vaginalis*	1	*Staphylococcus haemolyticus*	8
*Mobiluncus curtisii*	10	*Staphylococcus hominis*	3
*Olsenella uli*	1	*Staphylococcus lugdunensis*	3
*Slackia exigua*	2	*Staphylococcus pettenkoferi*	3
*Varibaculum cambriense*	7	*Staphylococcus simulans*	1
		*Staphylococcus *sp.	6
**Bacteroidetes**		*Staphylococcus warneri*	2
*Bacteroides coagulans*	8	*Streptococcus agalactiae*	4
*Bacteroides ureolyticus*	10	*Streptococcus anginosus group*	16
*Porphyromonas somerae*	6	*Streptococcus dysgalactiae*	1
*Prevotella bivia*	1	*Streptococcus oralis*	1
*Prevotella corporis*	4	*Streptococcus *sp.	4
*Prevotella disiens*	1		
*Prevotella *sp.	1	Possible novel species and genera*	
		TSWGenotypeA *Betaproteobacterium *[FM945400]	4
**Fusobacteria**		TSWGenotypeB *Porphyromonas *sp. [FM945401]	1
*Fusobacterium nucleatum*	1	TSWGenotypeC *Bacteroidetes *[FM945402]	3
*Fusobacterium *sp.	2	TSWGenotypeD *Clostridia *[FM945403]	5
		TSWGenotypeE *Clostridia *[FM945404]	2
**Proteobacteria**		TSWGenotypeF *Clostridia *[FM945405]	1
*Acinetobacter *sp.	1	TSWGenotypeG *Clostridia *[FM945406]	1
*Alcaligenes faecalis-like*	1	TSWGenotypeH *Bacilli *[FM945407]	2
*Escherichia coli*	7	TSWGenotypeI *Brevibacterium *sp. [FM945408]	2
*Klebsiella pneumoniae*	1		
*Proteus mirabilis*	1		

* accession number between brackets

We identified on average 8.6 species per woman (range 4–14). The species most often found were *Bacteroides ureolyticus *(n = 10 women), *Corynebacterium *sp. (n = 12), *Enterococcus faecalis *(n = 13), *Mobiluncus curtisii *(n = 10), *Staphylococcus epidermidis *(n = 19) and *Streptococcus anginosus *group spp. (n = 16). The neovaginal microflora of only one woman contained lactobacilli. *Neisseria gonorrhoeae *could not be not cultured.

There was no correlation between dilatation habits, having coitus, rinsing habits and malodorous vaginal discharge on the one hand and the presence of a particular species on the other hand. There was however a highly significant correlation between the presence of *E. faecalis *and sexual orientation: in heterosexual transsexual women (having a male partner) *E. faecalis *was present in 78.6% while it was only present in 14.2% of homosexual transsexual women and in 12.5% of bisexual transsexual women (p = 0.003). Equally there was a significant correlation between *E. faecalis *and the occurrence of regular coitus with a male partner: in those having regular coitus *E. faecalis *was present in 75% while in only 25% of those not having coitus (p = 0.027).

#### Detection by species specific PCR

DNA extracts of the 50 neovaginal samples were amplified with *16S rRNA *gene based primers specific for *A. vaginae, G. vaginalis *and *Mobiluncus curtisii*. Respectively 58% and 30% of the samples were PCR positive for *A. vaginae *and *G. vaginalis *(Table 2), with 24% of the samples positive for both species and 36% negative for both species.

**Table 2:** Amplification results with *A. vaginae *and *G. vaginalis *specific primers obtained for 50 neovaginal samples.

		*Gardnerella vaginalis*		
		
		+	-	Total
*Atopobium vaginae*	+	12	17	29
	
	-	3	18	21
	
	Total	15	35	50

The samples that were PCR positive for *G. vaginalis *were selected for amplification with bacterial vaginosis associated bacteria (BVAB) primers. All 15 specimens were PCR negative for BVAB1 and BVAB3 and only one specimen, positive for both *A. vaginae *and *G. vaginalis*, was PCR positive for BVAB2.

Remarkably, 41 of 50 neovaginal specimens showed an amplicon after amplification with *M. curtisii *primers (Table 3). Of these, 36 (88%) could be confirmed using *Mobiluncus *genus specific primers.

**Table 3:** Detection of *Mobiluncus curtisii *in 30 neovaginal samples: comparison between Gram stain, culture and species specific primers.

	C+P+	C+P-	C-P+	C-P-	Total
G+	6	0	6	1	13
G-	4	0	10	3	17

G+/-: Presence or absence of *Mobiluncus *cell types on Gram stain.

C+/-: Presence of absence of *M. curtisii *after anaerobic incubation on Columbia-based blood agar.

P+/-: Presence or absence of an amplicon after amplification with *M. curtisii *specific primers.

After amplification of the neovaginal DNA extracts with primers that target the ITS2-region of the rRNA cistron of Fungi and size determination of the amplified ITS2 by means of capillary electrophoresis, 6 specimens revealed an amplicon. Three specimens could not be sequenced and the remaining three sequences were identified as molds (resp. *Davidiella tassiana*, *Lycoperdon perlatum *and *Phaeosphaeria *sp.).

The PCR assay for *Chlamydia *on urine was negative for all participants.

## Discussion

The pH of the neovagina of the transsexual women in our study was consistently elevated (mean 5.8; range 5.0–7.0) as compared to that of the biological vagina. This is not unexpected as the acidic pH (3.8–4.5) of the vagina results primarily from lactic acid production by the resident lactobacilli [9,10] and is further enhanced through acidification by an active proton pump action of the vaginal epithelium – a mechanism upregulated by oestrogen [11]. In our patient series however, lactobacilli were consistently lacking, with only one transsexual woman with a penile skin-lined neovagina displaying some lactobacilli. As expected, and although these women show serum oestradiol levels comparable to those in substituted postmenopausal women, the environment of this penile skin-lined neovagina, does not support the growth of lactobacilli. This might be due to the absence of glycogen rich epithelial cells and to the absence of lactobacillus epithelial binding sites that are upregulated by oestrogen in the normal vaginal mucosa.

Our study indicates that the microflora of the neovagina is characterized by bacterial species from the skin and the intestinal microflora, somewhat similar to what is observed with premenarchal girls, who also lack a *Lactobacillus *dominated microflora, eliciting colonisation resistance. In particular, microscopy of Gram-stained neovaginal smears revealed a mixed microflora that was even more complex and denser than the one observed in biological women with bacterial vaginosis: we found mostly filamentous and fusiform shaped cells as well as *Mobiluncus *and *Spirochaetes *cell types. After culture on five different media a complex mixture of aerobe and (facultative) anaerobe species was found, with species usually found either on the skin and in the intestine or in the vagina of women with bacterial vaginosis.

Identification of the cultured isolates, by means of tDNA-PCR showed that the most abundant species of the neovaginal bacterial community included on the one hand species from the typical skin microflora, such as *S. epidermidis *and *S. anginosus *group spp., though not *S. aureus *which is usually prevalent on the perineal and vulvar skin, and on the other hand some typical intestinal species, such as *E. faecalis*, *M. curtisii *and *B. ureolyticus*. Interestingly, the latter three are also often present at low numbers in the vagina, with *E. faecalis *being associated with urinary tract infection and *M. curtisii *and *B. ureolyticus *being common to bacterial vaginosis.

It was recently suggested that the more complex the ecosystem changes are, as demonstrated by the presence of *Mobiluncus *and other anaerobes, the more difficult it is to cure bacterial vaginosis [12]. Therefore, the presence of *Mobiluncus*, known to have a high prevalence of resistance against metronidazole, indicates that additional treatment with clindamycin or amoxicillin might be useful in the case of a metronidazole resistant neovaginal infection in transsexual women [13,14].

*Enterococcus faecalis *was significantly and strongly associated with heterosexual orientation and penetrative sexual contact, indicating that the migration of this uropathogen to the vagina is strongly enhanced by intercourse, an observation that has previously been made for *E. coli *and *Enterococcus *species [15]. This finding is of importance to transsexual women's health as vaginal colonisation with uropathogens is generally known to precede urinary tract infection, while the neovagina presumably does not offer the colonisation resistance to such opportunistic pathogens observed among biological women with a lactobacilli-dominated microflora. This may explain at least in part why one in five transsexual women reported the frequent occurrence of dysuria.

At present it remains elusive to what extent other genito-urinary symptoms and complaints – both being rather common in our survey – among transsexual women can be attributed to microbiological factors. Frequent episodes of malodorous discharge were reported by one in four women and malodour was even more frequently observed upon gynaecological examination, which in turn might relate to the presence of faecal bacterial vaginosis-like microflora.

It should be acknowledged however that most of the cell types present in the Gram-stained smears could not be cultured by standard techniques. Hence, similar to other complex bacterial communities and the intestinal microflora [16] and the bacterial vaginosis microflora [6,7] in particular, much of the bacterial diversity of the neovaginal microflora – consisting of 8.6 cultivable species on average – remains uncharacterized through culture techniques. Still, using culture, twelve possibly novel species, designated TSW Genotypes A to L, were detected.

Specific assays were applied to detect fungal species in the neovagina, as well as to assess the presence of the index species in bacterial vaginosis. While *Candida albicans *was not seen on Gram stain, six women were found to harbour some fungal species, of which three remain unidentified. No firm conclusions can be drawn on the virtual absence of *Candida albicans*, however it may be acknowledged that in biological women *Candida *species are frequently encountered on the vaginal mucosa and that their colonisation of the vagina is stimulated by estradiol.

It is interesting to notice that the neovagina is colonized by largely the same intestinal species as the vagina in the absence of lactobacilli (some two thirds of transsexual women (64.0%) having evidence of colonisation with *G. vaginalis*, *A. vaginae*, or both, and over 80% of transsexual women harbouring *M. curtisii*) and thus that the same type of colonisation occurs, regardless the type of epithelium, vaginal mucosa of skin.

The BVAB species 1 to 3, belonging to the phylum *Clostridiales*, and BVAB2 in particular, were found to be highly specific for BV in biological women [17,18]. These were however not found in the neovaginal specimens, although it must be noted that the incidence of these species in the vaginal microflora of female patients of our hospital was quite low as well (unpublished data).

It is difficult to establish to what extent a bacterial vaginosis-like condition is present among these transsexual women. In a recent case report, a transsexual woman was diagnosed with bacterial vaginosis based on a Hay-Ison score of 2 (intermediate microflora) [19], although microscopy revealed numerous white blood cells and spirochaete-like organisms consistent with our observations. As a matter of fact, based on the criterion of a departure from a normal lactobacilli-dominated microflora, all patients in our study would qualify as having bacterial vaginosis-like microflora. We failed to document any association between specific bacteria and vaginal complaints – except for the unexplained and possibly spurious correlation between the presence of *Mobiluncus *and pain during sexual activity. It should be noted that also among biological women vaginitis symptoms are rarely associated with a single micro-organism.

The clinical significance of the very complex microflora of the penile-skin lined neovagina remains to be determined to a significant extent, which also means that we have few explanations for the high rates of vaginal complaints such as vaginal irritation and discharge in these patients. Furthermore, no proper advice can be given at present with regard to optimal vaginal hygiene in transsexual women, although douching with plain warm water has been suggested as an effective means to maintain the hygiene of the neovagina in transsexual women [20], but studies on this issue are actually lacking.

## Conclusion

This study is the first to describe the microflora of the penile skin-lined neovagina of transsexual women. The neovaginal microflora were devoided of lactobacilli and consisted of a mixed microflora of aerobe and anaerobe species usually found either on the skin, in the intestine or in bacterial vaginosis. Through tDNA-PCR we showed that the most abundant species of the neovaginal bacterial community included *S. epidermidis*, *S. anginosus *group spp., *E. faecalis, M. curtisii *and *B. ureolyticus*. Twelve possibly novel species, designated TSW Genotypes A to L, were detected. By using species specific PCR, we further established a particularly high prevalence of *A. vaginae*, *G. vaginalis *and *M. curtisii*. The clinical significance of the very complex microflora of the penile-skin lined neovagina remains to be determined however, and hence, at present we have few explanations for the high rates of vaginal complaints such as vaginal irritation and discharge in these patients. No proper advice can be given at present with regard to optimal vaginal hygiene in transsexual women.

## Methods

### Patient population

For the purpose of this study, 70 Dutch-speaking transsexual women who had a minimal interval of 6 months since SRS had been performed and who consulted one of the members of the gender team for treatment or follow-up during the year 2006 were invited to participate. After 4 weeks our target participation rate of 50 was reached and no further efforts were made to increase the sample size.

### Study procedures

This study complies with the recommendations of the Declaration of Helsinki and was approved by the Ethical Committee of our institution (Ghent University Hospital) under number 2006/375. Following written and oral informed consent, all women who agreed to participate completed the entire study protocol between March and June 2007.

Patients had been instructed to avoid sexual intercourse of any kind and to refrain from using vaginal hygiene products (soaps, lotions etc.) for at least three days prior to the examinations.

Upon enrolment the following items were enquired by the study nurse: medical and surgical history, sexual orientation, status of current relationship and the occurrence of frequent episodes (defined as once a month or more) of vaginal irritation and/or dysuria. All participants also filled in extensive questionnaires concerning general, mental and sexual health, of which the results were published earlier [21]. A fasting blood sample was taken to determine serum concentrations of estradiol and testosterone.

A speculum exam was performed by a gynaecologist. This speculum exam was performed using a 10 cm long and 2.5 cm wide Collins speculum. If introduction of this speculum was judged impossible or if the patient indicated that introduction was too painful, a more slender (2 cm wide) speculum was used. The speculum was only minimally lubricated with a few drops of sterile water. We refrained deliberately from the use of anything other than sterile water in order to avoid interference with the vaginal microflora. With the speculum in place, a cotton-tipped swab was rolled around against the mid-portion of one lateral wall to obtain a vaginal smear. The swab was then immediately smeared on a plain glass slide and allowed to dry at room temperature. A second sterile swab for culture and molecular analysis was rolled around against the same lateral wall of the mid-portion of the vagina and then placed into liquid Amies transport medium (Eswab, *Nuova Aptaca, Canelli, Italy*) and processed at the laboratory within 4 hours. Two commercial (nitrazine) pH strips were used to assess the pH of the neo-vagina: one with pH range from 1 to 10 (with accuracy of 1.0) and another one with pH range from 4 to 7 (with accuracy of 0.1) (*Merck, Darmstadt, Germany*). These strips were placed against the vaginal wall until sufficiently moistened and compared with the manufacturer's standard by a single observer (SW). In one patient insertion of the speculum was impossible due to an almost complete obliteration of the vagina. In this patient all vaginal swabs and pH-strips were taken superficially at the "introitus". *Chlamydia *was determined on a urine sample using a commercial real time PCR assay (Abbott RealTime CT, Abbott Laboratories, Illinois, US).

After completion of the study, we were left with some additional questions which we thought might be of influence on the vaginal microflora. Therefore the patients were sent an additional questionnaire in which they were asked about the regular use of vaginal hygiene products (once a month or more) and about the presence of bad-smelling vaginal discharge (once a month or more).

### Staining of slides

Smears were Gram stained (Mirastainer, Merck-Belgolabo, Overijse, Belgium) and examined under oil immersion at a magnification of 1000 by a single observer (GC).

### Culture and identification of cultured isolates by tDNA-PCR

For the first 30 women, 100 μl of liquid Amies transport medium was streaked onto 5 different agar plates upon arrival at the microbiology laboratory. The culture medium for recovering aerobic bacteria was Tryptic Soy Agar supplemented with 5% sheep blood (Becton Dickinson, Franklin Lakes, NJ). Staphylococci were recovered on Mannitol Salt Agar (Becton Dickinson, Franklin Lakes, NJ). Both media were incubated aerobically at 37°C for 24 h. The culture medium for cultivation of anaerobic bacteria was Columbia based agar with 5% sheep blood (Becton Dickinson, Franklin Lakes, NJ). MRS agar plates (Oxoid, Hampshire, UK) were used for the culture of lactobacilli. Both media were incubated anaerobically at 37°C for 5 days in an anaerobic workstation (BugBox Plus, LedTechno, Heusden-Zolder, Belgium). Selective GC-Lect Agar plates (Becton Dickinson, Franklin Lakes, NJ) for recovery of *Neisseria gonorrhoeae *were incubated in 5% CO_2 _atmosphere for two days. After incubation, all the isolates with different colony morphology were selected for identification. DNA was extracted by simple alkaline lysis: one colony was suspended in 20 μl of lysis buffer (0.25% sodium dodecyl sulfate-0.05 N NaOH), heated at 95°C for 15 min and diluted with 180 μl of distilled water. tDNA-PCR and capillary electrophoresis were carried out as described previously [22,23]. The isolates were identified by comparing their tDNA-PCR fingerprint with those of a library of tDNA-PCR fingerprints obtained from reference strains, using an in-house software program [22]. The library of tDNA-PCR fingerprints is available at http://allserv.ugent.be/~mvaneech/All_C.txt and the software can be obtained upon request.

### Sequencing of *16S rRNA *genes

Sequencing was carried out as described previously [7] and sequences were compared to the 16S rRNA sequences present in Genbank using BLAST. Sequences that had less than 98% similarity with previously known bacterial species were submitted to Genbank and were assigned accession numbers FM945400–FM945411.

### DNA extraction of vaginal swab samples

For DNA extraction from the dry vaginal swabs, 800 μl of NucliSens EasyMAG Lysis Buffer was added to 200 μl of liquid Amies transport medium, incubated for 10 min at room temperature and stored at -80°C until extraction was performed on the NucliSens EasyMag platform (BioMérieux, Marcy l'Etoile, France) according to the manufacturer's recommendations. DNA was eluted in 110 μl NucliSens EasyMAG Elution Buffer and DNA-extracts were stored at -20°C and were used for the purpose of species specific PCR.

### Species specific PCR for *Gardnerella vaginalis*

*G. vaginalis *species-specific primers (G_Z_), as designed by Zariffard *et al. *[24] were used. Briefly, a 20 μl PCR mixture contained respectively 0.05 μM primers, 10 μl of Promega master mix (Promega, Madison, WI), 2 μl of Easymag DNA-extract of the samples and distilled water. Thermal cycling with G_Z _primers consisted of an initial denaturation of 10 min at 94°C, followed by 50 cycles of 5 sec at 94°C, 45 sec at 55°C and 45 sec at 72°C, and a final extension of 10 min at 72°C. Five μl of the amplified product was visualized on a 2% agarose gel.

### Species specific PCR for *Atopobium vaginae*

A primer set ato167f (5' GCGAATATGGGAAAGCTCCG) and ato587r (5' GAGCGGATAGGGGTTGAGC) that allowed specific amplification of the 16S rRNA gene of *A. vaginae *was used as described earlier [7].

### Species specific PCR for BVAB

Species-specific PCR for bacterial vaginosis associated bacteria (BVAB1-3) was performed as previously described [17].

### Specific PCR for *Mobiluncus*

Genus-specific PCR for *Mobiluncus *spp. using primers Mob-s 5'GTGAACTCCTTTTTCTCGTGAA and Mob-as 5'CGCAGAAACACAGGATTGCATCC and species-specific PCR for *Mobiluncus curtisii *using primers Mob-V3 5'GCCAGCCTTCGGGGTGGTGT and Mob-V4 5'TCACGAGTCCCCGGCCGAACC was performed as described by Tiveljung *et al. *[25] with minor modifications. Briefly, a 20 μl PCR mixture contained 1 μM each of the primers, 10 μl of FastStart PCR master (Roche), 2 μl of Easymag DNA-extract of the samples and distilled water.

Thermal cycling consisted of an initial denaturation of 2 min at 94°C, followed by 35 cycles of 30 sec at 94°C, 30 sec at 60°C and 1 min at 72°C, with a final extension of 10 min at 72°C, and cooling to 10°C.

### Detection and identification of fungi using fluorescent fragment length analysis of the ITS2-PCR amplicon and sequencing

The amplification of the ITS2-region and subsequent capillary electrophoresis was performed as previously described [26,27].

Amplicons having a fragment length that was not present in the existing ITS2 library, which contains most of the clinically important yeast species, were sequenced as previously described [26].

### Data analysis

Distributions of continuous and discrete variables were summarized as means and standard deviations. Bivariate correlations are represented by Pearson's R if the observed distribution approximated a normal distribution, either by Spearman's rank correlation coefficient rho under the non-parametric assumption.

Statistical significance was accepted at the conventional two-tailed α = 0.05 significance level. All analyses were performed with the statistical software package SPSS 15.0 (Chicago, IlIinois).

## Abbreviations

BV: Bacterial Vaginosis; GID: Gender Identity Disorder; SRS: Sex Reassignment Surgery; WPATH: World Professional Association of Transgender Health; HBIGDA: Harry Benjamin International Gender Dysphoria Association; VMF: Vaginal Microflora; DNA: Deoxyribonucleic Acid; PCR: Polymerase Chain Reaction; RNA: Ribonucleic Acid; TSW: Transsexual Women; BVAB: Bacterial Vaginosis Associated Bacterium; ITS2: Internal Transcribed Spacer 2.

## Authors' contributions

SW conceived the study and its design, gathered the data, and drafted the manuscript. HV participated in the interpretation of the data, performed statistical analysis and helped to draft the manuscript. JG and SM both participated in the design of the study and revised it critically. GL, BS, and EDB performed the laboratory analysis and contributed to the interpretation of the data. GC performed the Gram-staining and its analysis and critically revised the manuscript. MV helped in the interpretation of the data and critically revised the manuscript. RV performed the laboratory analysis, helped in the interpretation of data and the drafting of the manuscript. All authors read and approved the final manuscript.
